# Supplementary material for: A Comprehensive Assessment of Ultraviolet-Radiation-Induced Mutations in Flammulina filiformis Using Whole-Genome Resequencing
Source: J Fungi (Basel). 2024 Mar 20;10(3):228. doi: 10.3390/jof10030228 (PMC10971301; doi:10.3390/jof10030228)
Supplement: Supplementary file 1 [file jof-10-00228-s001.zip › Supplementary Material S8/KEGG annotation/out/64381550635650.os/KO/out_map/map04142.html]

KEGG PATHWAY: Lysosome - Reference pathway


|  |  |
| --- | --- |
| **Lysosome - Reference pathway** |  |

[
Pathway menu
| Organism menu
| Pathway entry
| Show description
| User data mapping
]

Lysosomes are membrane-delimited organelles in animal cells serving as the cell's main digestive compartment to which all sorts of macromolecules are delivered for degradation. They contain more than 40 hydrolases in an acidic environment (pH of about 5). After synthesis in the ER, lysosomal enzymes are decorated with mannose-6-phosphate residues, which are recognized by mannose-6-phosphate receptors in the trans-Golgi network. They are packaged into clathrin-coated vesicles and are transported to late endosomes. Substances for digestion are acquired by the lysosomes via a series of processes including endocytosis, phagocytosis, and autophagy.

|  |  |
| --- | --- |
| Reference pathway | 100% |
